# Vascular Dysfunction in Colorectal Cancer: Scoping Review of Current Evidence for Guiding Future Research

**DOI:** 10.1007/s12029-026-01438-6

**Published:** 2026-03-21

**Authors:** Sydney R. DeJonge, Noah G. DuBose, Gerald Gantt, Lisa Tussing-Humphreys, Robert W. Motl

**Affiliations:** 1 Department of Kinesiology and Nutrition, College of Applied Health Sciences, University of Illinois Chicago, Chicago, IL USA; 2 University of Illinois Cancer Center, University of Illinois Chicago, Chicago, IL USA

**Keywords:** Colorectal cancer, Vascular function, Flow-mediated dilation, Pulse wave velocity

## Abstract

**Purpose:**

We conducted a scoping review of research on vascular function (VF) in colorectal cancer (CRC) survivors and developed a research agenda to address current limitations.

**Methods:**

We applied the Preferred Reporting Items for Systematic Reviews and Meta-Analysis Scoping Review (PRISMA-ScR) when conducting and reporting this review. We searched 3 databases (PubMed, EBSCOhost, and EMBASE) in October 2025 for publications on VF and vascular dysfunction (VD) that included measures of arterial stiffness and endothelial function in CRC survivors. There were 348 manuscripts identified for title and abstract screening, and 341 were excluded for not meeting the inclusion criteria.

**Results:**

Our search and subsequent screening identified seven papers reporting measures of VF in CRC survivors. Five papers focused on the impact of systemic therapies on measures of VF, including PWV and FMD, whereas two papers examined the effect of colon surgery on surrogate measures of VF. Increased arterial stiffness and atherosclerosis were observed following systemic treatment; however, endothelial function was inconsistent. Following colon surgery, endothelial function declined, while arterial stiffness remained unchanged.

**Conclusion:**

CRC survivors who underwent systemic treatment had increased arterial stiffness and atherosclerosis, and those who underwent colon surgery had endothelial dysfunction. Overall, there is limited research on VF in CRC survivors, and we outline an agenda for future research to investigate VF in this population and address the numerous gaps in the field.

**Implications for Cancer Survivors:**

This review highlights that CRC survivors may experience vascular health risks following systemic therapy and colon surgery, emphasizing the need for improved VF monitoring and survivorship care.

## Introduction

Cancer and cardiovascular disease (CVD) remain the leading causes of death among adults in the United States (US) [1]. Colorectal cancer (CRC) is the third most common cancer diagnosed annually in both men and women, and it accounts for approximately 9% of all cancer-related deaths in the US [1]. Advances in CRC screening, early detection, and treatment have improved survival rates, yet CRC survivors (i.e., all people who have been diagnosed with CRC) often experience adverse health outcomes following diagnosis and treatment, including multiple comorbidities, fatigue, pain, reduced physical function, and diminished quality of life [2].

CRC survivors often present with traditional CVD risk factors at diagnosis (i.e., older age, obesity, physical inactivity, smoking, and alcohol consumption), and roughly 40% of CRC survivors have between one and three comorbid conditions, with 20% having four or more [3]. The most common comorbidities in CRC survivors involve circulatory and cardiovascular systems and include hypertension, chronic obstructive pulmonary disease, type 2 diabetes mellitus, and ischemic heart disease [3]. The combination of traditional risk factors and comorbidities yields a higher likelihood of CVD-related mortality among CRC survivors than those without cancer, and the risk of CVD increases substantially (~ 45%) in the first two years following a CRC diagnosis [4].

Cardiotoxicity damages the cardiovascular system, including the heart and blood vessels, during or after cancer treatment [5]. Cardiotoxicity is associated with chemotherapy, radiation, and newer advanced immunotherapies and targeted treatments [5–7]. Cardiotoxicity occurs in approximately 40% of CRC survivors and is associated with heart failure, hypertension, and myocardial ischemia [8]. An underlying feature of cardiotoxicity is impaired vascular function (VF) [9]. Measuring VF in CRC survivors following diagnosis and treatment is of substantial importance, as VF outcomes (e.g., pulse-wave velocity, flow-mediated dilation) serve as indicators of cardiovascular damage and CVD risk [6, 7]. Monitoring of VF and vascular dysfunction (VD) would enable focal management of cardiovascular complications in CRC survivors through pharmacological and non-pharmacological interventions (e.g., physical activity, diet) that improve and support cardiovascular health.

To date, there has been a limited but emerging focus on VF and its measurement in CRC survivors. Based on prior literature, there are decrements in VF following primary CRC treatments (e.g., 5-fluorouracil, bevacizumab), yet it is unknown to what extent these vascular changes persist after treatment. With this scoping review, we aim to provide a landscape of current research and a roadmap that guides future research on VF/VD in CRC survivors. There is a pressing need to address this gap, as there are CRC survivors who may be living with VD and more emergent cardiovascular complications, and by assessing VF, we may be able to better understand and limit VD and subsequent complications in CRC survivors.

## Methodology

There are multiple types of reviews, and a scoping review describes and synthesizes evidence on a specific body of literature, providing a map of the volume, nature, and scope of an existing research area. We opted for a scoping review rather than a systematic review (i.e., a summary of the effectiveness of an intervention based on specific outcomes), as it provides a comprehensive overview of the heterogeneity of VF in CRC and identifies gaps in the literature [10, 11]. Scoping reviews have become increasingly popular in recent years for clinical research in chronic disease populations. We have applied the Preferred Reporting Items for Systematic Reviews, and Meta-Analysis Scoping Review (PRISMA-ScR) evidence-based approach when conducting and reporting this review paper [12], , and the final protocol was registered with the Open Science Framework (https://osf.io/fg5wu/overview). This scoping review provides an overview of [1] VF and outcome measures [2], VF in CRC survivors and identifying gaps in the literature [3], identifying limitations and groups at higher risk of VD [4], call to action, and [5] advocating for future research.

### Search Strategy and Data Selection

**Fig. 1:**
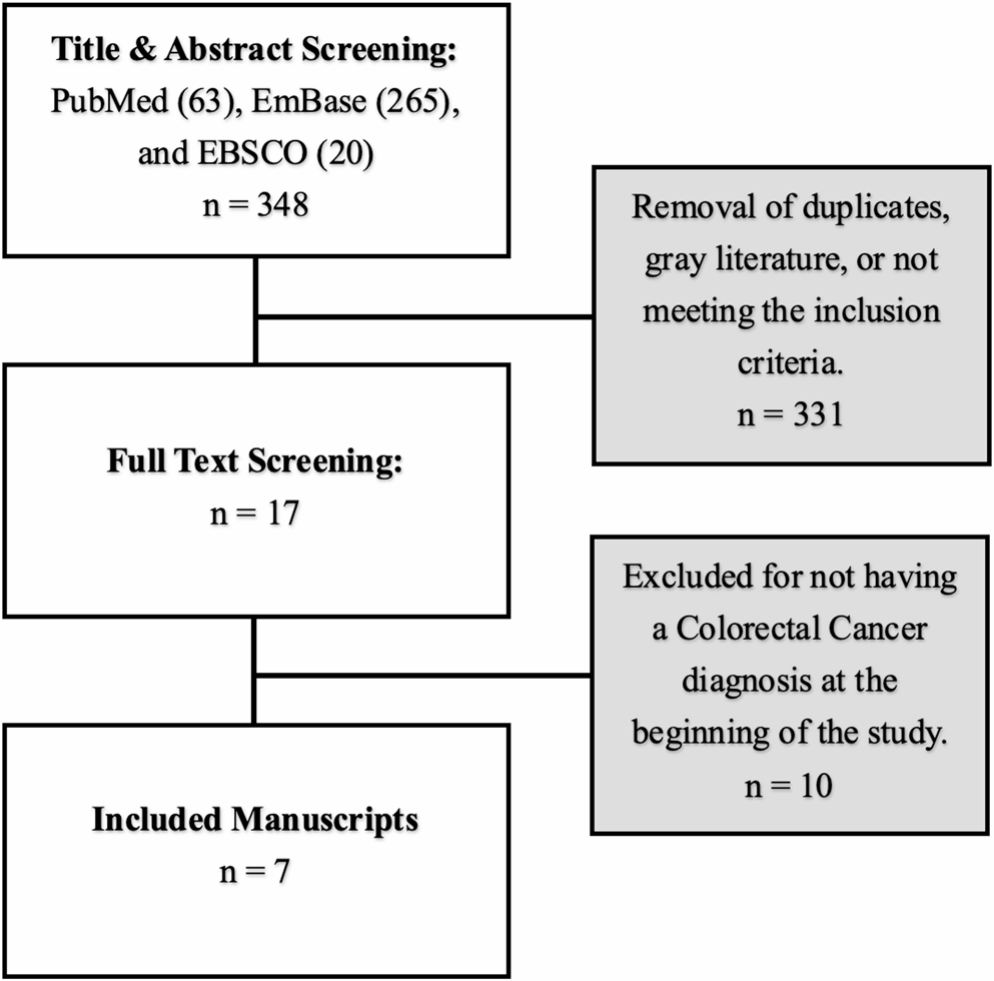
PRISMA-ScR Consort Flow Diagram

Our protocol adhered to PRISMA-ScR guidelines [12]. Three electronic databases (PubMed, EBSCOhost, and EMBASE) were searched to identify and retrieve published studies on October 16, 2025. The search strategy combined relevant terms of “colorectal cancer” and “vascular function” (e.g., pulse pressure, arterial stiffness, arterial compliance, endothelial function, flow-mediated dilation, etc.) and was created in collaboration with a health sciences librarian. All search terms are presented in Table 1 and were adapted for each database; the full Boolean search query for each database is provided in Table 1. SRD completed the title and abstract screening. Duplicate studies and gray literature were removed, and the remaining papers were thoroughly reviewed by SRD and NGD with the following criteria: a) a manuscript published in English, (b) included one sample of any stage colorectal cancer survivors (i.e., mixed samples accepted), and (c) included a measure of VF listed in Table 1. Discrepancies from full article screening were further discussed with RWM and LTH. Figure 1 provides a PRISMA-ScR flow diagram of the review process. The papers that fit our scope were selected for inclusion in this scoping review, and all others were excluded. The included papers are presented in Table 2.

**Table 1:**
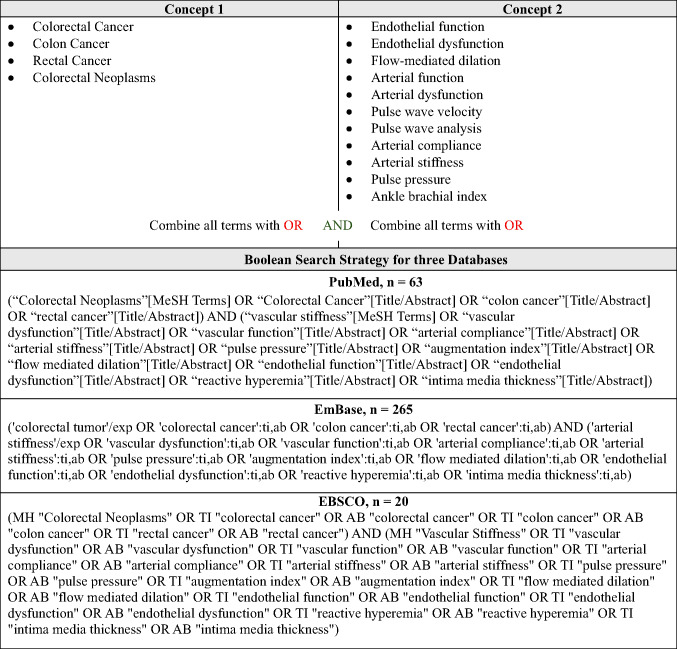
Concepts and Literature Search Strategy

### Vascular Function: Definition, Importance, and Measurements

VF is defined as the homeostatic mechanism responsible for maintaining tissue perfusion and the appropriate exchange of gases and metabolic byproducts that are fundamental for sustaining life. Impairments in vascular homeostatic mechanisms manifest as functional and structural changes, including reductions in endothelial vasoactive substances and compositional shifts in the arterial wall. These changes result in decreased arterial compliance and regulatory control, collectively defined as VD.

VF is commonly assessed using noninvasive measures of flow-mediated dilation (FMD) and pulse wave velocity (PWV) in large central arteries (i.e., the aorta) and peripheral conduit arteries (i.e., the brachial artery). Surrogate measures of VF are often collected as they are more feasible and include pulse pressure, augmentation index (AIx), peak blood flow, reactive hyperemia index, carotid intima-media thickness (cIMT), and correlates of VF, such as arterial blood pressure.

FMD is a technique that examines an arterial segment, most often the brachial artery, using ultrasonography before, during, and after 5 min of occlusion with a blood pressure cuff (e.g., brachial occlusion). FMD involves changes in vessel diameter and is reported as percent dilation from baseline to peak diameter by examining the increase in blood flow immediately following release of the brachial occlusion [13]. This measure focuses on endothelial nitric oxide-dependent vasodilation of vascular smooth muscle, which is necessary for satisfying the metabolic demand of muscular work following occlusion. FMD is recognized as a prognostic indicator of future cardiovascular events, and this relationship is stronger in disease populations [14]. We hypothesize that FMD could serve as a significant measure of CVD risk in CRC survivors.

PWV is the gold standard measure of arterial stiffness. This non-invasive measure assesses pulse transit time between blood pressure waves between two pulse locations, oftentimes the carotid and femoral arteries (carotid-femoral PWV [cfPWV]), highlighting the aortic segment of the arterial tree [15]. PWV can be measured between the brachial and ankle arteries (brachial-ankle PWV [baPWV]), emphasizing stiffness of the central and peripheral arteries. PWV is driven by a vital function of the aorta, namely, dampening pulsatile blood flow from the heart before it enters the periphery and low-pressure organs (i.e., the brain, kidneys, etc.), where insufficiently dampened pressure can result in microvascular injury [16]. Therefore, a stiffer aorta results in higher PWV and faster pulse transit speed. The reciprocal of arterial stiffness is arterial compliance, which is reported as a measure of the elasticity of an arterial segment. When combined with other CVD risk factors (e.g., atherosclerosis, hypertension, pulse pressure), PWV can improve CVD prediction [17], and we hypothesize that it may serve as a beneficial marker in CRC survivors.

## Key Findings

We identified seven papers that examined the impact of CRC treatment (i.e., systemic therapies and surgery) on the structural and functional aspects of the vasculature in CRC survivors (Table 2). Five papers highlight the effects of CRC-related systemic therapies, and the CRC patient samples were overall largely male, over the age of 59 years, and had a stage II, III, or IV diagnosis. Two papers focus on the effects of CRC-related surgeries, and the CRC patient samples were overall largely female, between the age of 30 and 79 years, and had any stage of CRC (e.g., 0 through IV). The vascular outcome (e.g., FMD and PWV) results included in those studies have strong relevance for CVD and may guide the understanding of heightened risk for CVD following CRC-related treatment.

### Vascular Dysfunction in CRC Survivors who Underwent Systemic Therapies

The first study investigated the reversibility of vascular dysfunction after completion of bevacizumab treatment (a vascular endothelial growth factor [VEGF] inhibitor) [18]. The sample was comprised of 14 patients with metastatic CRC (*n* = 12) and breast cancer (*n* = 2). Vascular outcomes, including FMD, cfPWV, and capillary density, were assessed at baseline (7 days prior to the first administration), after 6 weeks (before the third weekly administration), and at least 3 months after the last treatment administration. The overall results between baseline to 6-weeks post treatment, there were significant reductions in FMD (6.3% to 3.2%, *p*<.01) and capillary density (18.2 n^c^ to 13.3 n^c^, *p*<.001, and significant increases in cfPWV (8.9 m/s to 9.6 m/s, *p*<.05). However, when examining a subsample (*n* = 8) who completed a second follow-up, FMD and capillary density significantly reduced between 6-weeks and 3-months following baseline (*p*<.05, and *p*<.001, respectively), but not cfPWV (*p*>.05). This study reported the capillary density was reversible 3-months post treatment (*p*<.001); however, this was not the case for FMD or PWV as these values remained unchanged from 6-weeks to 3-months post treatment (*p*>.05). There was evidence of VD following 6-weeks of bevacizumab treatment and it remained for 3-months. Conversely, capillary density did not return to baseline at the 3-month timepoint (*p*<.001).

The second study examined changes in cIMT, a surrogate measure of VF that quantifies the thickness of the intima and media layers of the carotid artery, along with other atherosclerosis risk factors, in patients (*n* = 58) with metastatic CRC (*n* = 47) and renal cell carcinoma (*n* = 11) who had undergone VEGF inhibitor treatment [19]. Ultrasonography was applied for cIMT assessment, defined as the difference between the blood-intima and media-adventitia interfaces on the posterior wall of the carotid artery. Compared with baseline (0.70 mm), the metastatic patients had increased in cIMT thickness at 3 months (0.76 mm), 6 months (0.76 mm), and 12 months (0.83 mm) following treatment (*p*<.05). These data suggested VEGF inhibitor treatment rapidly increases laboratory measures of atherosclerosis in these patients with metastatic disease, and this has likely implications of VD.

The next study examined arterial stiffness in patients (*n* = 171) with metastatic CRC (*n* = 93), kidney (*n* = 60), and gastrointestinal stromal tumors (*n* = 18) before and after chemotherapy [20]. CRC survivors received between six and eight panitumumab (an epidermal growth factor receptor [EGFR] inhibitor) combination with oxaliplatin, irinotecan, or capecitabine treatment cycles, and the vascular outcomes of PWV and AIx were assessed before and immediately after chemotherapy that lasted on average seven weeks. In CRC survivors, there were significant changes in systolic blood pressure (SBP) (137.3 vs. 133.5 mmHg, *p*<.01), AIx corrected for 75 bpm (AIx75) (17.2% vs. 21.8%, *p*<.001), cfPWV (7.6 vs. 8.4 m/s, *p*<.001), and carotid-radial PWV (crPWV) (7.7 vs. 8.6 m/s, *p*<.001). The differences in AIx75 (*p*<.001), cfPWV (*p*<.05), and crPWV (*p*<.001) remained when adjusting for factors such as sex, body mass index (BMI), and blood pressure (BP). Importantly, it was noted that cfPWV had the greatest increase following irinotecan-based treatment (*n* = 28) compared with capecitabine- or oxaliplatin-based treatments. Overall, the data suggest that on average, seven weeks of targeted treatment for metastatic CRC may lead to evidence of VD.

Another study investigated the impact of adjuvant 5-fluorouracil (5FU) and oxaliplatin on vascular, cardiac, neurovascular, and physical function in patients with colon cancer [21]. The 5FU/leucovorin group (*n* = 12) received 24–30 weeks of chemotherapy, and the 5FU/leucovorin and oxaliplatin group (*n* = 16) received 24 weeks of chemotherapy. Femoral artery FMD was assessed based on published techniques [13], and peak muscle blood flow of the forearm and leg arteries was measured using venous occlusion plethysmography [22]. These outcomes (reported as mean (standard error)) were evaluated both before and after treatment. In the 5FU/leucovorin group, FMD decreased 2.7% (11.7 (2.3)% to 9.0 (1.6)%), whereas the change was smaller in the 5FU/leucovorin and oxaliplatin group (i.e., 0.8% (8.5 (1.5)% to 7.7 (1.7)%). Importantly, the changes in both groups were not statistically significant. There further were no significant changes in forearm (2.8(SE = 0.4) to 2.6(SE = 0.3 both groups) and leg blood flow; 5FU/leucovorin: (2.4(SE = 0.3) to 2.4(SE = 0.4) mL/min/100mL) and 5FU/leucovorin and oxaliplatin: (2.3(SE = 0.3) to 2.8(SE = 0.2) mL/min/100mL) peak blood flow in both groups. The data suggest no implications for VD following completion of these forms of chemotherapy that aim to disrupt DNA synthesis and cause cell death.

The last study examined the effects of adjuvant chemotherapy on indices of arterial stiffness in patients with CRC [23]. One group (*n* = 16) completed 6 cycles of FOLFOX (5FU and oxaliplatin) treatment, and the second group (*n* = 54) completed 6 cycles of XELOX (oxaliplatin and capecitabine tablets) treatment. There were significant changes in indices of arterial stiffness, including AIx75 increasing from 16.9% to 21.3% (*p*<.001), crPWV increasing from 7.2 to 8.2 m/s (*p*<.01), and cfPWV increasing from 7.5 to 8.3 m/s (*p*<.001). The changes remained significant when examined overall and by subgroup (*p*<.001). Overall, the data suggest evidence of VD in patients with CRC after 6 cycles of treatment and further suggest that these changes are acute rather than chronic and independent of treatment type.

### Vascular Dysfunction in CRC Survivors who Underwent Colorectal Surgery

The first study assessed the impact of colon surgery on endothelial function using digital pulse amplitude tonometry (EndoPat2000) in 31 patients with stages I to IV colon cancer [24]. Three surgeries were examined in this study: sigmoid resection (*n* = 11), right colon resection (*n* = 19), and subtotal colectomy (*n* = 1). Digital pulse amplitude tonometry was quantified as the reactive hyperemia index (RHI [%]), measured at baseline, occlusion, and hyperemia on both index fingers, with one finger serving as the control. The RHI is a ratio of hyperemia to baseline measures, expressed as a percentage. All patients underwent preoperative and postoperative RHI measures at 4 h, 1 day, and 2 days. All patients had normal preoperative RHI values (mean: 1.86%), and there was a significant decrease in RHI at the 2-day postoperative measure (mean: 1.65%, *p*<.05), suggesting evidence of VD, specifically endothelial dysfunction, following major colon cancer surgery.

The second study examined radial pulse pressure (RPP) in 30 patients with stage 0-IV CRC before and after colectomy [25]. RPP was measured at three locations on both wrists (Chun, Guan, and Chy; Huand-T1 Sphygmograph) with the patients in a supine position. Values of augmentation index (AIx) were further derived from the RPP waves. Vascular measurements were taken before and within 24 h after the colectomy. There were no significant differences in RPP across all three locations pre- and post-colectomy; however, a substantial decrease in AIx was observed, specifically in the right distal location (Chy) (*p* < .05). These results indicate that VD was not present following a colectomy in this sample of CRC survivors.

**Table 2:** Summary of Study Characteristics, Treatment Exposures, Measurements, and Vascular Outcomes

Systemic Therapies						
Paper	Patients	Age (years; mean ± SD, unless specified)	Sex (Male/ Female)	Vascular measurement	Treatment Exposure	Change with Exposure
Steeghs et al., 2010 [18]	Overall: *n* = 22 Metastatic CRC: *n* = 19	59–61(range) Metastatic CRC: NR	Overall: 10/12 Metastatic CRC: NR	FMD cfPWV Capillary density	Bevacizumab dosage: 7.5 mg/kg/3 weeks	*Overall Results*: *Baseline to 6-weeks post treatment*: ↓ FMD* ↑ cfPWV* ↓ Capillary density** *6-weeks post treatment to 3-months post treatment*: ↔ FMD ↔ cfPWV ↑ Capillary density** *Overall Repeated Measures*: ↓ FMD* ↑ cfPWV ↑ Capillary density**
Studentova et al., 2016 [19]	Overall: *n* = 58 Metastatic CRC: *n* = 47	Overall: 62 ± 8 Metastatic CRC: NR	Overall: 37/21 Metastatic CRC: NR	cIMT	CRC (*n* = 44) - Bevacizumab: 5 mg/kg/30 mins) + folinic acid: 50 mg bolus + fluorouracil: 400 mg/m^2^ bolus + 2400 mg/m^2^ infusion for 46 h+ oxaliplatin: 130 mg/m^2^/2 hours. CRC (*n* = 2) - Bevacizumab: 5 mg/kg/30 mins) + folinic acid: 50 mg bolus + fluorouracil: 400 mg/m^2^ bolus + 2400 mg/m^2^ infusion for 46 h+ oxaliplatin: 130 mg/m^2^/2 hours + irinotecan: 180 mg/m^2^/90 mins. CRC (*n* = 1) - Bevacizumab: 5 mg/kg/30 mins) + folinic acid: 50 mg bolus + fluorouracil: 400 mg/m^2^ bolus + 2400 mg/m^2^ infusion for 46 h+ oxaliplatin: 100 mg/m^2^/2 hours	*Overall Results*: *Baseline to 3-months post treatment*: ↑ cIMT** *Baseline to 6-months post treatment*: ↔ cIMT** *Baseline to 12-months post treatment*: ↑ cIMT*
Res et al., 2018 [20]	Overall: *n* = 171 Metastatic CRC: *n* = 93	Overall: 62.9 ± 10.7 Metastatic CRC: 65.2 ± 10.7	Overall: 124/47 Metastatic CRC: 71/22	cfPWV crPWV AIx75	Metastatic CRC: - Panitumumab (*n* = 93), 8 (3–8) cycles, 530 (250–900) mg - Oxaliplatin (*n* = 44), 8 (4–8) cycles, 190 (140–480) mg - Irinotecan (*n* = 28), 8 (6–8) cycles, 343 (240–410) mg - Capecitabine (*n* = 21), 6 (3–8) cycles, 1300 (200–2000) mg	*Overall Results: NR* *Metastatic CRC Results*: *Baseline to Post Chemotherapy*: ↓ SBP* ↑ cfPWV** ↑ crPWV** ↑ AIx75** *Baseline to Post Oxaliplatin Treatment*: ↓ SBP* ↑ cfPWV** ↑ crPWV** ↑ AIx75** *Baseline to Post Irinotecan Treatment*: ↓ SBP ↑ cfPWV** ↑ crPWV** ↑ AIx75** *Baseline to Post Capecitabine Treatment*: ↓ SBP ↑ cfPWV** ↑ crPWV** ↑ AIx75**
Groehs et al., 2020 [21]	Overall, Stages II – III CRC: *n* = 29	Overall, Stages II – III CRC: 60 ± 1	Overall, Stages II – III CRC: 14/15	FMD Peak blood flow	5 FU Group (*n* = 12): - 5FU: 370 mg/m^2^ + leucovorin: 50 mg/m^2^ IV weekly, 24–30 weeks 5 FU + Oxaliplatin Group (*n* = 17): - 5FU: 500 mg/m^2^ + leucovorin: 20 mg/m^2^ IV weeks 1–6, & oxaliplatin 85 mg/m^2^ on weeks 1, 3, 5, every 8 weeks for 3 cycles (total 24 weeks).	*Overall Results*: ↓ FMD ↔ peak blood flow *Baseline to Post 5-FU Treatment*: ↓ FMD ↔ peak blood flow *Baseline to Post 5 FU + Oxaliplatin Treatment*: ↓ FMD ↔ peak blood flow
Visvikis et al., 2020 [23]	Overall, Stages II – III CRC: *n* = 70	Overall, Stages II – III CRC: 64.9 ± 10.5	Overall, Stages II – III CRC: 55/15	cfPWV crPWV AIx75	FOLFOX (*n* = 16) - Oxaliplatin: 85 mg/m^2^ + leucovorin: 200 mg/m^2^ + 5FU bolus: 400 mg/m^2^ & 48 h 5FU infusion of 1200 mg/m^2^ XELOX (*n* = 54) - Oxaliplatin: 130 mg/m^2^ + Xeloda 2gr/m^2^ oral tablets for 14 consecutive days.	*Overall Results*: ↑ SBP** ↑ cfPWV** ↑ crPWV* ↑ AIx75** *Baseline to Post FOLFOX Treatment*: ↑ SBP** ↑ cfPWV** ↑ crPWV* ↑ AIx75** *Baseline to Post XELOX Treatment*: ↑ SBP** ↑ cfPWV** ↑ crPWV** ↑ AIx75**

Colorectal Surgery						
**Paper**	**Patients**	**Age (years)**	**Sex (Male/** **Female)**	**Vascular Measurement**	**Surgery Exposure**	**Change with Exposure**
Ekeloef et al., 2017 [24]	Overall, Stages 0 – IV CRC: *n* = 31	Overall, Stages 0 – IV CRC: 67 (95% CI’s: 63–71)	Overall, Stages 0 – IV CRC: 18/13	RHI	Sigmoid resection (*n* = 11) Right colon resection (*n* = 19) Total colectomy (*n* = 1)	*Preoperative to Postoperative Day 2 Results*: ↓ RHI*
Chuang et al., 2020 [25]	Overall, Stages 0 – IV CRC: *n* = 30	Overall, Stages 0 – IV CRC: 30–79 (range)	Overall, Stages 0 – IV CRC: 11/19	RPP AIx	Colectomy	*Preoperative to Postoperative Results*: ↔ RPP ↓ AIx*

*CRC* Colorectal Cancer, *cfPWV*carotid-femoral pulse wave velocity, *FMD* flow mediated dilation,*NR* not reported, *cIMT*carotid intima media thickness, *crPWV* carotid-radial pulse wave velocity, *AIx75* augmentation index corrected to 75 beats per minute, *AIx* augmentation index, *SBP* systolic blood pressure, *RHI* reactive hyperemia index, *RPP*radial pulse pressure

Change with Exposure is indicated with ↑ (increase), ↓ (decrease), or ↔ (no change) per vascular measurement. Significance of Change:* *p*<.05 and ** *p*<.001

## Discussion

CRC survivors are at a heightened risk for CVD based on their pre-existing traditional risk factors (e.g., older age, obesity, physical inactivity, smoking, and alcohol consumption) and comorbidities (e.g., type 2 diabetes mellitus, hypertension, etc.) [3, 4]. With that, it is necessary to understand how VF outcomes are assessed in this population and how treatment affects vascular health, given the strong association between CRC and later CVD events.

Based on the summary of evidence, it is suggested that acute VD is present following systemic treatments for CRC survivors, including targeted therapies and chemotherapies. VD in these patients manifests as increased arterial stiffness and atherosclerosis [18–20, 23]. However, there were inconsistent findings in measures of endothelial function following systemic treatments [18, 21]. These findings collectively suggest that even a short period of treatment is deleterious to vascular health. There was inconsistent evidence of VD following colon surgery. Whereas markers of endothelial function were impaired, indices of arterial stiffness remained unaffected [24, 25]. Future research should also examine VF following various treatments, including systemic therapy and surgery. As more data are accrued, we will gain valuable insight into how the vasculature is affected and may, in turn, inform prevention and treatment of VD.

## Limitations within VF in CRC Research

We identified several major limitations in research on VF in CRC survivors. First, there is a significant discrepancy in the examination and reporting of VF outcomes across racial and ethnic groups. For example, there was minimal reporting of race and ethnicity in these papers, which limits the generalizability of the results. We further know that American Indians and Alaska Natives and non-Hispanic Black males are at the highest risk for CRC incidence and mortality [1]. These racial groups have the highest prevalence of hypertension and CVD, which could be associated with a higher risk of VD outcomes [26, 27]. Second, there is a lack of consistency in the instruments used to measure VF in CRC. For example, PWV measures reported in this review were assessed across different arterial segments (e.g., cfPWV vs. crPWV), and only 2 studies included FMD outcomes. Additionally, following colon surgery, only surrogate measures of VF were assessed, rather than the gold-standard measures of PWV and FMD. Consistency across measurements will facilitate easy comparison of VF among CRC survivors and other populations, including controls or other cancer survivors. Third, compiling a comprehensive search strategy was challenging due to the limited scope of this body of literature. For example, some aspects of VF are poorly defined, leading to a wide variety of terms, definitions, and instrumentation for this topic. Lastly, there were issues and inconsistencies that interfered with the interpretation of the results, including: (a) no control groups (i.e., healthy controls without cancer), (b) variable timepoints between cycles of treatment or surgery, (c) vascular function measurement methodology, and (d) lack of control for confounding factors such as blood pressure that would directly impact PWV outcomes.

## Call to Action

Based on the results and limitations presented in this scoping review, there is a call to action to include measures and outcomes of VF in CRC survivors. There is emerging evidence that VD is prevalent in CRC survivors who have a high prevalence of comorbidities and CVD (e.g., Non-Hispanic Blacks) [1, 28]. This implies an immediate call to action from researchers and clinicians to implement more non-invasive measures of VF into their routine assessments for CRC survivors. There is further potential to explore VD management in this population, with lifestyle factors. This may involve developing and implementing targeted interventions that include exercise training and lifestyle health behaviors (e.g., physical activity and diet) to restore or preserve vascular integrity in CRC survivors, and this should be a top priority for future research. The prioritization of randomized controlled trials and longitudinal studies can help clarify mechanisms, identify high-risk groups, and explore multimodal approaches for vascular rehabilitation. The studies should reflect diverse demographic, socioeconomic, and treatment backgrounds across the cancer continuum. By including VF in CRC survivor research, we may reduce the burden of CVD and improve health-related quality of life.

## Future Research

Our scoping review highlights limited data on assessments of VF/VD in CRC survivors. With that, numerous gaps in the literature exist, demonstrating clear opportunities for advancements in this research niche. We have identified multiple areas of research that will extend the existing literature on VF, including its measurement, consequences, and lifestyle interventions among CRC survivors (Table 3). These areas of research include [1] measurements of VF [2], VF in the context of CRC and demographic and clinical characteristics [3], outcomes related to VF in CRC [4], management of VD in CRC, and [5] mechanisms behind VD in CRC.

**Table 3:** Research topics, issues, questions, and significance for advancing the understanding of vascular function in CRC survivors

Topic	What is/are the issues?	Research Questions	Significance
1. Measurement of Vascular Function (VF) in Colorectal cancer (CRC)	There are minimal data on non-invasive measures of VF (e.g., FMD, PWV, AIx75, etc.) in CRC survivors, and most of these outcomes have been measured in the brachial and carotid arteries. This highlights a gap in assessment of other segments of the arterial tree (e.g., popliteal artery, cerebral arteries).	• Is there a difference between CRC survivors and controls in VF, including measures of arterial stiffness and endothelial function? • Is there a difference between VF outcomes in upper- vs. lower- extremities following cancer treatment? • Is there a difference in cerebral blood flow and VF between CRC survivors and controls?	Examining VF in different segments of the arterial tree may highlight the mechanisms behind VD in CRC following cancer treatment. This research will create a foundation of differences between CRC patients and controls, helping to identify specific consequences in VF following treatment. These outcomes can further help discover other primary locations for measurement.
2. VF in CRC survivors: Demographic and Clinical Characteristics	VF has been examined in a small sample of CRC survivors; however, these assessments have not included control patients without cancer. There is also limited data across demographic and clinical characteristics in CRC survivors.	• Are there differences in VF among all the stages of CRC (e.g., 0-IV), and how do these outcomes compare to controls? • How does VD vary with treatment type (e.g., chemotherapy, radiation, surgery, immunotherapy, etc.) and time since completed treatment? • How does VF/VD compare among younger adults with CRC vs. older adults with CRC? • Does VF/VD differ among racial and ethnic populations? • How does VF compare among adults with different social determinants of health and socioeconomic status?	Examining VF throughout the cancer continuum, and among different cancer stages would be foundational for clinicians and researchers in identifying how we can best serve our patients. Similarly, understanding how the different types of cancer treatment will shed light on how VF/VD impact symptoms and other negative consequences in CRC. Lastly, examining different ages, sexes, races, ethnicities, and socioeconomic factors among CRC will be insightful to target strategies to improve/maintain VF/VD in each of these populations specifically.
3. Outcomes of Vascular Dysfunction (VD) in CRC survivors (e.g., symptoms, cardiorespiratory fitness, physical function, cognition, etc.)	There are many negative consequences following a CRC diagnosis and treatment (e.g., depression, poor health related quality of life, cognitive impairment, etc.), yet little is known about the interaction between VF/VD and these outcomes in CRC survivors.	• Does VF/VD influence health related quality of life and physical function in CRC survivors? • What is the relationship between VF/VD and clinical symptoms (e.g., depression, anxiety, fatigue, etc.) in CRC survivors? • Is VF/VD associated with cognition function in CRC survivors following completion of treatment? • What are the effects of time restricted eating, Mediterranean diet, or other dietary interventions on VF in CRC survivors?	Understanding the relationship between VF/VD and outcomes/negative consequences following cancer treatment may signify VF as a target for research and managing outcomes in CRC such cognitive impairment and poor physical function.
4. Management of VD in CRC survivors (e.g., physical activity, exercise, diet, sleep, etc.)	Currently, there is limited research on management of VD in CRC survivors. There is a need to examine the impact of cardiorespiratory fitness, and lifestyle behaviors such as physical activity, sedentary behavior, diet, sleep and more on VD in CRC survivors.	• Does physical activity serve as a beneficial lifestyle behavior for improving VF in CRC? • Does physical activity and exercise during treatment influence VF/VD in CRC? • What is the impact of sleep on VF/VD outcomes in CRC during and following treatment?	CRC survivors are wanting to improve many outcomes following cancer and treatment, and this research will help guide the future of lifestyle behavior interventions for CRC.
5. Mechanisms of VD in CRC survivors (e.g., structure and function of the vasculature, autonomic function, coagulation, etc.)	There is limited research on mechanisms of VD in CRC survivors. Specifically, there is little research on the role of autonomic function and how it relates to the vascular structure and function in this population. Exploring other aspects such as coagulation and fibrinolytic potential could highlight the prevalence of thrombosis development following cancer treatment.	• Examine the long-term impact of systemic therapy on the vasculature structure and function. • Examine changes in coagulation and fibrinolytic potential following cancer treatment, and how these factors impact VF in CRC. • What is the influence and reduced autonomic function correlate with VF/VD in CRC? How does this impact other outcomes in this population? • How does reduced autonomic function impact the structure and function of the vasculature?	Mechanistic research is essential in understanding VD in CRC survivors. Identifying the role of autonomic function and other factors such as coagulation and fibrinolytic potential can help explain VD following cancer and its subsequent treatment.

Note.* CRC *Colorectal Cancer,* VF *Vascular function,* VD V*ascular dysfunction

## Conclusion

This scoping review identified a limited body of research on VF in CRC survivors, thereby providing numerous opportunities for future research. Evidence suggests increased arterial stiffness and the presence of atherosclerosis in CRC survivors following various forms of systemic treatment, yet there were inconsistencies in the outcomes of endothelial function. In contrast, following colon surgery, declines in endothelial function were observed, whereas measures of arterial stiffness remained unchanged. Overall, there is a need for future research to clarify inconsistencies, strengthen evidence on VD in CRC survivors, and prioritize approaches for mitigating and reversing VD.

## Data Availability

No datasets were generated or analysed during the current study.
